# The Promotion of Cell Proliferation by Food-Derived Bioactive Peptides: Sources and Mechanisms

**DOI:** 10.3390/metabo15080505

**Published:** 2025-07-29

**Authors:** Yuhao Yan, Yinuo Liu, Xinwei Zhang, Liting Zan, Xibi Fang

**Affiliations:** 1 Laboratory of Nutrition and Functional Food, College of Food Science and Engineering, Jilin University, Changchun 130062, China; 2 College of Animal Science, Jilin University, Changchun 130062, China; 3 Linhai Special Agricultural Products Technology Extension Station, Linhai 317000, China

**Keywords:** peptides, functional food, cell repair and proliferation, osteoporosis

## Abstract

Cell proliferation plays a pivotal role in multiple physiological processes, including osteoporosis alleviation, wound healing, and immune enhancement. Numerous novel peptides with cell proliferation-promoting activity have been identified. These peptides exert their functions by modulating key cellular signaling pathways, thereby regulating diverse biological processes related to cell proliferation. This work summarizes peptides derived from animals and plants that stimulate cell proliferation, focusing on their amino acid composition, physicochemical properties, and preparation techniques. Furthermore, we highlight the major signaling pathways—such as the PI3K/Akt, MAPK/ERK, and Wnt/β-catenin pathways—that have been implicated in the mechanistic studies of food-derived peptides. Through the analysis and summary of previous studies, we observe a notable lack of in vivo animal models and clinical trials, indicating that these may represent promising directions for future research on food-derived bioactive peptides. Meanwhile, the potential safety concerns of proliferation-enhancing peptides—such as immunogenicity, appropriate dosage, and gastrointestinal stability—warrant greater attention. In summary, this review provides a comprehensive overview of the sources and mechanisms of cell proliferation-promoting peptides and addresses the challenges in industrializing bioactive peptide-based functional foods; therefore, further research in this area is encouraged.

## 1. Introduction

In recent years, it has been widely recognized that food proteins provide a variety of bioactive peptides. These peptides are known for their functional and nutritional benefits. These peptides could be released by enzymatic hydrolysis, solid-phase peptide synthesis, fermentation, and other methods. Apart from providing essential amino acids and repairing tissue, these peptides were shown to exhibit diverse functions, including the promotion of cell proliferation [1,2]. Cell proliferation is a fundamental process that plays a crucial role in tissue and organ growth, development, repair, osteoporosis prevention, wound healing, and immune enhancement. In eukaryotic organisms, cell division occurs either when they attain a specific size or in response to external signals such as growth factors or hormones [3]. This process involves an intricate regulatory mechanism wherein the activation of crucial pathways and the involvement of pivotal functional genes are essential.

Osteoporosis has become a significant health concern for middle-aged and elderly individuals. According to the Chinese Osteoporosis Epidemiological Survey, osteoporosis affects more than 200 million individuals worldwide, and the projected expenditure for managing osteoporotic fractures is anticipated to exceed USD 25 billion by 2025 [4]. Osteoporosis primarily results from decreased osteoblast activity, leading to reduced bone mass [5] and the deterioration of bone structure [6], which increases the risk of fractures in middle-aged and elderly individuals. Osteoblasts are critical for bone development, remodeling, and repair [7]. Peptides from food sources could promote osteoblast proliferation and differentiation via the phosphoinositide 3-kinase (PI3K)/protein kinase B (AKT) signaling pathway [8], mitogen-activated protein kinase (MAPK) signaling pathway [9], and Wnt/β-catenin signaling pathway [7]. Therefore, a feasible approach to prevent osteoporosis is to stimulate osteoblast activity through the supplementation of food-derived peptides.

Cell proliferation is essential for wound healing. The skin serves as a barrier, protecting internal organs and tissues against external damage, such as mechanical injuries and metabolic dysfunction [10,11]. Skin injuries need effective healing to ensure health and survival. Skin wound healing has four stages: hemostasis, inflammation, proliferation, and tissue remodeling [12].

Cell proliferation is a key stage. Neovascularization, tissue regeneration, and remodeling depend on the proliferation and migration of fibroblasts and endothelial cells. These processes are essential for forming the extracellular matrix (ECM) and promoting angiogenesis [13]. Thus, enhancing the proliferation of fibroblasts and endothelial cells facilitates the regeneration of damaged skin [12]. Numerous bioactive peptides from food sources have been found to possess the ability to facilitate human skin fibroblast (HSF) and human umbilical vein endothelial cell (HUVEC) proliferation to promote wound healing via the MAPK [3], AKT [12], and nuclear factor κ-B (NF-κB) signaling pathways [14].

Cell proliferation is also essential in boosting immunity. The immune system protects the body from pathogens, toxic antigens, and tumor cells [15]. An abnormal immune response can lead to excessive inflammation and tissue damage [16]. Lymphocytes play a crucial role in fighting infections and monitoring cellular mutations [17]. Activated macrophages help eliminate pathogens, dead cells, and foreign bodies through phagocytosis [18]. Peptides from food sources promote the proliferation of macrophages and lymphocytes via the NF-κB and MAPK signaling pathways. These peptides also reduce the secretion of pro-inflammatory cytokines like TNF-α, IL-6, NO, and PGE2, thereby offering anti-inflammatory and immunomodulatory effects [19,20,21].

Peptides promoting cell proliferation have gained significant attention in recent research. The literature search in this study was retrieved from the Web of Science database (time frame: 1900 to 31 December 2024) and screened based on preset inclusion and exclusion criteria. Ultimately, 97 studies were included. Furthermore, this work summarizes peptides from food that promote cell proliferation, focusing on their mechanisms, safety risks, and comparison of their abilities. These findings provide a foundation for further research on functional foods and nutritional health products in humans.

## 2. Source and Preparation of Cell Proliferation-Promoting Peptides

### 2.1. Cell Proliferation-Promoting Peptides Derived from Aquatic Animals

In recent years, many studies have focused on peptides in aquatic products. Marine bioactive peptides are a rich source of diverse bioactive compounds. Studies over the past decades have provided sufficient evidence on their production, optimization, characterization, composition, and sequence. Many peptides from aquatic animals promote osteocyte proliferation and alleviate osteoporosis. Peptides from sea cucumbers have the strongest cell proliferation capacity [3]. Most of these peptides are derived from deep-sea fish and shellfish, with sea cucumbers having the most robust cell proliferation capacity [12]. However, the cell proliferation capacity of peptides extracted from the scales of *Mozambique tilapia* is weak [22].

Among the peptides derived from aquatic animals (Table 1), the longest peptide is the peptide NINECFSSPCENOGICODEIDGYNCVCOPGFTGTHCE from sea cucumber [3], while the shortest peptide is KSA from fish bone [23]. Most peptides have 4 to 20 amino acid residues, indicating that the length of peptides may significantly affect cell proliferation. Peptides promoting bone development often contain glutamic acid, glutamine, aspartic acid, glycine, arginine, and leucine, which support osteoblast metabolism and bone growth [24]. The activation of the ERK1/2, AKT, PI3K/AKT, MAPK, and SMAD pathways in osteoblasts is triggered by receptors like LRP1, IGF-1R, and TGF-β in response to peptides [25]. These signaling cascades are crucial for osteoblast proliferation, migration, survival, differentiation, and matrix formation [26].

The peptide extraction process from aquatic animals involves several steps: pre-treatment, enzymatic disintegration, purification, and identification. Different pre-treatments are used for various foods. For example, collagen peptides extracted from sturgeon cartilage, such as *Coryphaena hippurus* and *Nibea japonica*, require the removal of non-collagen with NaOH and fat with orthopedic methods [14,27]. Tissues obtained from oysters and *Johnius belengerii* were first crushed [9,23]. The main enzymes used in the enzymatic disintegration process include pepsin, alcalase, neutral protease, papain, and trypsin [9,28]. Peptides are purified by ultra-performance liquid chromatography separations [9,29], and filtration and ultra-centrifugation [27]. Amino acid composition is analyzed using an amino acid analyzer. For example, to obtain peptides from *Johnius belengerii* bone, the fish bone powder is digested with pepsin in a 5% acetic acid solution (pH 2.0) for 48 h, followed by filtration, centrifugation, and purification using fast protein liquid chromatography. The molecular mass and sequence of the purified peptide are determined with a quadrupole time-of-flight (TOF) mass spectrometer coupled with electrospray ionization [23].

In short, about 48% peptides derived from aquatic animals can promote osteoblast growth and differentiation, especially short peptides with high activity [30]. Compared to other protein sources, peptides derived from aquatic animals have a higher essential amino acid content and are easily digestible. Enzymatic hydrolysis is the main method for extracting these peptides. Before hydrolysis, non-collagen components and lipids must be removed. The peptides are then purified and identified using filtration, centrifugation, and HPLC-MS/MS for further study. Aquatic animals are rich in nutrients, and their peptides show strong potential to promote cell proliferation. This potential deserves more research.

**Table 1:** Cell proliferation-promoting peptides derived from aquatic animals.

Sequence	Source	Cell/Animal Model	Amount Added	Action Pathway	Cell Performance	Animal Performance	References
YRGDVVPK	Oyster	Mouse embryo osteoblast precursor cell (MC3T3-E1)	100 nM	MAPK signaling pathway	Cell proliferation and differentiation ↑	Osteoporosis ↓	[9]
TPERYY	Tilapia scale	MC3T3-E1 cell	109 μg/mL	Wnt/β-catenin signaling pathway	Cell proliferation, differentiation, and mineralization ↑	Bone health ↑ Osteoporosis ↓	[7]
KSA	*Johnius belengerii*	MC3T1-E1 cell	—	MAPK signaling pathway	Cell proliferation and differentiation ↑	Osteoporosis ↓ bone formation ↑	[23]
YPRKDETGAERT	*Mytilus edulis*	MC3T3-E1 cell		Bone morphogenetic protein type 2 (BMP-2) signaling pathway	Cell proliferation and differentiation ↑	Femoral ↑, osteoporosis ↓	[31]
SCIH (28 peptides)	Sea Cucumber Intestine	MC3T3-E1 cell	25 μg/mL	Wnt/β-catenin signaling pathway	Cell proliferation and differentiation ↑	Bone growth ↑	[29]
RPQYPQYPS, LSFSPY	Sea cucumber	MC3T3-E1 cell	100 μg/mL	—	Proliferation and mineralization ↑	Osteoporosis ↓	[32]
FDNEGKGKLPEEY, FWDGRDGEVDGFK VLQTDNDALGKAK IVLDSGDGVTH, MVAPEEHP	*Pinctada martensii*	MC3T3-E1 cell	2 μg/mL	—	Cell proliferation and differentiation ↑	Osteoporosis ↓	[33]
WSMP	Oyster shells	MC3T3-E1 cell	100 μg/mL	BMP-2 signaling pathway	Cell proliferation and differentiation ↑	Osteoporosis ↓	[34]
MNKKREAEFQ	*Gadus morhua*	MC3T3-E1 cell	100 μg/mL	BMP/WNT signaling pathway	Cell proliferation and differentiation ↑	Osteoporosis ↓	[35]
—	*Chanos chanos*	Human osteosarcoma cell (MG-63)	100 μg/mL	—	Cell proliferation and differentiation ↑	Osteoporosis ↓	[36]
—	*Mytilus coruscus*	Mouse Mononuclear Macrophage cell (RAW264.7)	100 μg/mL	MAPK signaling pathway	Cell proliferation ↑, the phagocytosis of cells ↑	Immunomodulation ↑	
—	*Nibea japonica*	Mouse Embryonic Fibroblast cell (NIH-3T3)	25 μg/mL	NF-κB signaling pathway	Cell proliferation and migration ↑	Wound healing ↑	[14]
—	*Sipunculus nudus*	HUVEC, Human immortalized epidermal cells (HaCaT), HSF cell	—	—	Cell proliferation ↑	Wound healing ↑, scar formation ↓	[28]
VTPY, VLLY	Sea cucumber	HSF cell and HUVEC cell	1000 nmol/mL	ERK/AKT signaling pathway	Cell proliferation ↑	Wound healing ↑	[12]
NINECFSSPCEN OGICODEIDGYN CVCOPGFTGTHCE	Sea cucumber	Human melanoma cell	10 nM	MAPK and AKT signaling pathway	Cell proliferation ↑	Wound healing ↑	[3]
QIGFIW, IGIGPSGAS	Bigbelly seahorse	Mouse myoblast cell (C2C12)	100 μg/mL	P38MAPK/AKT signaling pathway	Cell proliferation and differentiation ↑	Skeletal muscle differentiation and endurance ↑	[37]
VGRTNSH	Oyster	Human normal breast cell (MCF-10A)	50 μg/mL	PRL/AKT/STAT5 and Mammalian target of rapamycin (mTOR)/Ribosomal protein S6 kinase B1 (S6KB1) signaling pathway	Cell proliferation ↑	Lactation ↓	[38]
—	*Coryphaena hippurus*	bone marrow-derived macrophage cell (BMMS)	50 ng/mL	MAPK signaling pathway	Cell proliferation and differentiation ↑	Osteoporosis ↓	[27]
—	*Mozambique tilapia*	Human dermal papilla cells (hDPC)	62.5 ppm	Wnt/β-catenin signaling pathway	Cell proliferation ↑	Hair growth in the back skin tissue ↑	[22]
MGLAGPR, MGDVLNF, EAPLMHV, TEAPLMHV, TEAPLMHV	Octopus	Mouse mammary epithelial cell (HC11)	25 μg/mL	—	Cell proliferation ↑	The synthesis of β-casein ↑	[39]

↑: increase; ↓: decrease; —: reported but not fully confirmed.

### 2.2. Cell Proliferation-Promoting Peptides Derived from Plants

Some plant-derived peptides have been found to promote cell proliferation. These peptides primarily originate from dicotyledons, and approximately 90% contain 6 to 12 amino acid residues (Table 2). The longest peptide identified is the peptide SKWQHQQDSCRKQGVNLTPCEKHIMEKIQGRGDDDDDDDDD from seeds [40], while the shortest is LRW from pea [8]. Hydrophobic amino acids in these peptides may help them interact with cell membranes and reduce inflammation [41]. Sequence analysis shows that most peptides contain polar uncharged amino acids, except for di- and tripeptides. Glycine and glutamine are the most common, while cystine and threonine are rare. Peptides with alkaline or hydrophobic amino acids at the N-terminal are linked to immune-regulating effects [42].

Peptides are prepared using various methods, including alkali extraction, acid precipitation, and ultra-sound-assisted acid or enzyme extraction. The main enzymes are pepsin, alcalase, neutral protease, papain, and trypsin [20,43]. The peptides in *Porphyra haitanensis* are extracted using a chemical method [44]. The peptides are purified and identified using reversed-phase high-performance liquid chromatography [21], gel permeation chromatography [45], and ultra-performance liquid chromatography with tandem mass spectrometry [20]. Most plant peptides promote immune cell proliferation and regulate immunity. The peptide from rice bran protein shows the strongest effect, significantly increasing HUVEC cell proliferation at 1 μM after 72 h. It also reduces wound area in a concentration-dependent manner below 10 μM. [46]. Mung bean hydrolysates have the weakest effect. They exhibit activity only at 200 mg/mL, but can still reduce the release of inflammatory factors and regulate inflammation in vitro [47].

In summary, the amino acid composition of the peptide has a great impact on its anti-inflammatory effect, especially the presence of hydrophobic amino acids. Enzymatic hydrolysis and ultra-sound-assisted extraction are the main methods for extracting peptides from plants. After extraction, the peptides are purified and identified using various chromatography techniques. It provides candidate materials for the development of trauma repair drugs.

**Table 2:** Cell proliferation-promoting peptides derived from plants.

Sequence	Source	Cell/Animal Model	Amount Added	Action Pathway	Cell Performance	Animal Performance	References
IQDKEGIPPDQQR	Extruded Lupin	RAW 264.7 cell	1 μg/mL	MARK signaling pathway	Cell proliferation ↑	Inflammatory response ↓	[20]
—	Mung bean	RAW264.7 cell	200 mg/mL	—	Cell proliferation ↑, phagocytosis ↑	Immunomodulation ↑ and anti-inflammation	[47]
YGPSSYGYG	*Pseudostellaria heterophylla*	RAW264.7 cell	200 μg/mL	Toll-like receptors (TLR)/NF-κB/TNF-αsignaling pathway	Cell proliferation ↑, the endocytosis of macrophages ↑	Immunomodulation ↑	[21]
SSFSKGVQRAAF	Rice bran	HUVEC cell	1 μM	—	Cell proliferation and migration ↑	Wound healing ↑	[46]
DIGGL	*Ulva prolifera*	HUVECs cell	100 μM	—	Cell proliferation ↑	Immunomodulation ↑ blood pressure ↓	[43]
LRW	Pea	MC3T3-E1 cell	50 μM	PI3K/AKT, AKT/Runx2 signal pathway	Cell proliferation, migration, differentiation, and mineralization ↑	Osteoclast formation and the prevention of osteoporosis ↓	[8]
DEDEQIPSHPPR	Soybean	MC3T3-E1 cell	70 μM	MAPK signaling pathway	Cell proliferation, differentiation, and mineralization ↑	Osteoporosis ↓	[48]
—	Zein peptides	C2C12 cells	200 μg/mL	Mechanistic Target of Rapamycin Complex 1/2 (mTORC1/mTORC2) signaling pathway	Cell proliferation and cell cycle progression ↑	Sarcopenia ↓	[49]
NQLDQMPR, PVNKPGRFE and the other 52 peptides	Soybean	Rat small intestine crypt epithelial cell (IEC-6)	1 mg/mL	—	Cell proliferation ↑	Intestinal inflammation ↓	[45]
—	*Porphyra haitanensis*	IEC-6 cell	100 μg/mL	—	Cell proliferation and migration ↑	Intestinal epithelial wound healing ↑	[44]
—	*Cornus officinalis*	Chicken Embryonic Fibroblasts (CEF)	0.4 mg/mL	—	Cell proliferation ↑	Free radicals ↓, anti-oxidation	[50]
SKWQHQQDSCRKQGVNLTPCEKHIMEKIQGRGDDDDDDDDD	Seed peptide	Male C57BL/6Jnarl mice, EL-4 T cell	—	—	Cell proliferation ↑, cytokines ↑	Anti-inflammatory, antioxidant ↑	[40]

↑: increase; ↓: decrease; —: reported but not fully confirmed.

### 2.3. Cell Proliferation-Promoting Peptides Derived from Livestock Products

Peptides that promote cell proliferation have been identified from livestock products, mainly from milk proteins and collagen (Table 3). Peptides from bovine lactoferrin show the strongest activity [51]. Peptides from duck and chicken meat are 3–4 amino acids long [6,52], while the length of peptides that are derived from porcine bones and milk is between 8 and 10 [18,53]. The longest peptide is HHGDQGAPGAVGPAGPRGPAGPSGPAGKDGR from bovine bone collagen hydrolysates [54], and the shortest peptide is the peptide EF, from black-bone silky fowl [52]. These peptides usually contain hydrophobic and acidic amino acids, but cysteine and glutamine are rare. Their preparation methods are similar to those used for aquatic peptides. Raw materials are crushed and hydrolyzed using enzymes like trypsin, pepsin, papain, and bromelain [55]. After centrifugation and filtration, the peptides are purified using molecular exclusion chromatography [52] and reversed-phase high-performance liquid chromatography [56]. The purified peptides are identified with HPLC-MS/MS [51,52,55]. In short, most peptides from animal products are medium-sized and mainly extracted from collagen-rich bones. The raw materials are crushed and then hydrolyzed with enzymes. After centrifugation and filtration, peptides are purified using molecular exclusion chromatography and UPLC. Peptide composition is identified by HPLC-MS/MS, which helps discover more effective peptide sequences.

Seafood-derived peptides mainly promote osteoblast proliferation and help relieve osteoporosis. Plant-derived peptides mainly stimulate immune cell proliferation, showing anti-inflammatory and immune-regulating effects. Cell proliferation is the basis of these functions. In the following section, we discuss the mechanisms by which peptides promote cell proliferation.

## 3. Cell Proliferation-Promoting Mechanism of Peptides

### 3.1. Signal Pathway

**Figure 1:**
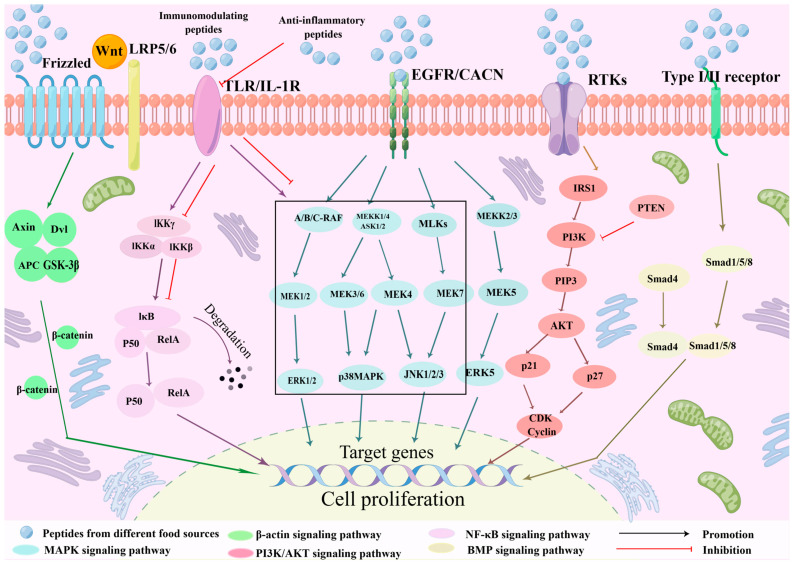
The main pathways for peptides promote cell proliferation.

The proliferative function of peptides has been investigated in various cell types, including MC3T3-E1, NIH-3T3, RAW264.7, HaCaT, human colonic epithelial, lymphocytes, and fibroblasts. The signal pathways in MC3T3-E1, NIH-3T3, RAW264.7, and HaCaT cells have been extensively studied. Here, we provide a summary of these pathways (Figure 1).

#### 3.1.1. MAPK Signaling Pathway

The MAPK signaling pathway is the most extensively explored among the studies of peptides that promote cell proliferation. This pathway plays a crucial role in mediating various physiological functions in the body. MAPK is a cytoplasmic protein kinase that activates transcription factors through a conserved cascade (MAPKKK-MAPKK-MAPKMAPK) when stimulated [59]. The MAPK signaling pathway has been identified as crucial for the regulation of pro-inflammatory mediators, cytokines, and the expression of inflammatory proteins [60]. It is known that MAPKs, including the p38 MAPK signaling pathway, c-Jun N-terminal kinase (JNK) signaling pathway, and the ERK signaling pathway, serve as important mediators in controlling bone formation [61]. The p38 and ERK1/2 MAPK signaling pathways enhance osteoblast differentiation and proliferation via phosphorylating Runx2, while the JNK signaling pathway exerts its function through other critical factors such as activator protein 1 (AP-1) and β-catenin [26]. Peptide KSA, derived from fish bone peptide, stimulates the phosphorylation of all MAPK members, including the p38 MAPK signaling pathway, ERK signaling pathway, and JNK signaling pathway. It promotes the proliferation and differentiation of osteoblasts [23].

The MAPK signaling pathway plays a role in promoting wound healing. Peptides QIGFIW and IGIGPSGAS have a potential effect of upregulating key myogenic regulatory proteins, including myogenic differentiation antigen (MyoD), myogenin (MyoG), and myosin heavy chain (MyHC) at concentrations of 100 μg/mL and 50 μg/mL, respectively, in the pot-bellied hippocampus. Additionally, P38 MAPK, the main MAPK signaling pathway, could activate MyoD gene expression [37].

#### 3.1.2. Wnt/β-Catenin Signaling Pathway

The Wnt pathway is critical in regulating skeletal development and homeostasis [62]. Specifically, the Wnt/β-catenin signaling pathway is vital for controlling the proliferation and differentiation of osteoblasts [63]. Activation of this pathway involves binding Wnt protein to Frizzled receptors on the cell surface, which in turn bind to low-density lipoprotein receptor-related protein 5/6 (LRP5/6) receptors. The binding inhibits the activity of glycogen synthase kinase 3 beta (GSK-3β), preventing the phosphorylation of β-catenin and ensuring its stability [64]. Unphosphorylated β-catenin is transported into the nucleus, where it enhances the transcriptional activity of T cell factor/lymphoid enhancer factor family (TCF/LEF) and increases the expression of osteogenic genes [7]. Peptide GPAGPPGPIGNV, which is derived from yak bone collagen, has been found to stimulate the upregulation of β-catenin mRNA and its protein levels. It also activates transcriptional markers such as β-catenin, Wnt5a, and Frizzled-5, which play a significant role in the Wnt/β-catenin signaling pathway, thereby promoting the proliferation and differentiation of osteoblasts [56]. The peptide VSEE, extracted from desalted duck egg white, has been demonstrated to enhance osteoblast proliferation through the activation of the Wnt/β-catenin pathway [6].

#### 3.1.3. NF-κB Signaling Pathway

NF-κB is essential for normal immune and cellular functions. In resting macrophages, NF-κB dimers are bound to the inhibitor IκB and remain inactive in the cytoplasm. Upon stimulation, IκB is phosphorylated, allowing NF-κB to enter the nucleus and activate gene transcription [65]. The NF-κB protein serves as a significant regulator in both innate and adaptive immune responses [66], accelerating cell proliferation, preventing apoptosis, promoting cell migration, invasion, and inducing angiogenesis and metastasis [67]. Furthermore, the activation of NF-κB swiftly and briefly responds to viral and bacterial infections, necrotic cell debris, oxidative stress, DNA damage, and pro-inflammatory cytokines [68]. The peptide YGPSSYGYG activates RAW264.7 cells by increasing NO secretion, enhancing pinocytosis, and raising ROS and TNF-α levels [21]. Earlier investigations have shown a strong correlation between wound healing and the NF-κB signaling pathway [14]. *Nibea japonica* collagen peptides promote the proliferation of fibroblasts through the NF-κB pathway, highlighting their potential role in enhancing wound healing. After treatment with different concentrations of *Nibea japonica* collagen peptides, the levels of NF-κB p65, IκB kinase α (IKKα), and IκB kinase β (IKKβ) in cells exhibited a notable dose-dependent increase. Additionally, protein levels of specific growth factors (such as epidermal growth factor (EGF), fibroblast growth factor (FGF), and vascular endothelial growth factor A (VEGF)) were elevated in NIH-3T3 cells [14].

#### 3.1.4. PI3K/AKT Signaling Pathway

The PI3K/AKT signaling pathway is essential for regulating bone metabolism in osteoblasts and osteoclasts. It is closely linked to the MAPK, MEK/ERK, and NF-κB pathways [69]. It plays a significant role in maintaining human bone homeostasis. However, when the PI3K/AKT pathway is activated, it can lead to an increase in the expression of the genes associated with inflammation through various downstream targets, with NF-κB being identified as a principal regulator [70]. Upon stimulation by receptors, particularly cytokine receptors, the membrane protein PI3K can induce AKT phosphorylation directly or indirectly, leading to the subsequent activation of NF-κB [71]. The classic PI3K signaling pathway consists primarily of components such as the PI3K family, phosphatidylinositol-3,4,5-trisphosphate (PIP3) gene, phosphatase and tensin homolog (PTEN) gene, and AKT gene. PIP3, as an important second messenger in this pathway, plays a role in activating the pathway [72]. The PTEN gene triggers the dephosphorylation of PIP3, which is produced by PI3K signaling. By reducing the level of activated AKT, it prevents the downstream signaling of AKT, thereby inhibiting the activation of the PI3K signaling pathway [73]. PTEN serves as an important antagonistic gene in this pathway. AKT binds to PIP3, gets activated, and regulates cell function by phosphorylating various downstream kinases [74]. Collagen peptides sourced from porcine bone have been shown to enhance proliferation and prevent apoptosis in osteoblasts through the activation of the PI3K/AKT pathway [55]. The peptide NINECFSSPCENOGICODEIDGYNCVCOPGFTGTHCE extracted from sea cucumber can induce protein docking through GRB2-associated binder 1 (GAB1) to activate PI3K, which converts phosphatidylinositol 4,5-bisphosphate (PIP2) to PIP3, allowing AKT phosphorylation before activating pyruvate dehydrogenase kinase 1 (PDK1). An increase in AKT gene expression may lead to the phosphorylation of NF-κB, which is crucial for cell survival. Additionally, AKT can phosphorylate other substrates, such as glycogen synthase kinase-3 (GSK-3), contributing to the inhibition of cyclin D production when its levels are sufficiently elevated [3].

#### 3.1.5. BMP Signaling Pathway

The bone morphogenetic protein (BMP) signaling pathway is involved in inducing the differentiation of mesenchymal stem cells (MSCs) in the bone marrow into osteocytes, as well as promoting the proliferation of osteoblasts and chondrocytes. BMPs are a well-explored category of functional proteins that can be utilized to delay or treat osteoporosis [75]. These proteins are crucial for maintaining bone density and can stimulate the differentiation of bone marrow-derived mesenchymal stem cells (BMSCs) into osteoblasts, thus increasing the number of mature osteoblasts and enhancing their differentiation capabilities [76]. BMP exerts its effect on osteoblasts by activating BMP membrane receptors and their signaling pathways [77]. Antioxidant peptides derived from blue mussels, specifically PIISVYWK and FSVVPSPK, are known to enhance the proliferation of osteoblasts as well as the expression of bone morphogenetic proteins-2/4 (BMP-2/4). These peptides lead to the upregulation of mothers against decapentaplegic homolog 1/5 (Smad1/5) phosphorylation and various transcription factors such as Runx2 and osterix [33]. The levels of bone morphogenetic protein 2/4 (BMP-2/4) expression in osteoblasts significantly increased when treated with the peptide YPRKDETGAERT derived from *Mytilus edulis*. This peptide also upregulates type I collagen, p-Smad1/5, and transcription factors, including osterix and Runx2, in mesenchymal bone marrow-derived MSCs, promoting osteoblast proliferation and alleviating osteoporosis [31].

We have identified and summarized five signaling pathways through which peptides facilitate cell proliferation. These pathways are crucial in promoting the growth and differentiation of osteoblasts and can also modulate downstream signaling pathways to alleviate osteoporosis. The MAPK signaling pathway and NF-κB signaling pathway contribute to wound healing by promoting fibroblast proliferation and the expression of related proteins. The NF-κB signaling pathway also plays a significant role in reducing inflammation and enhancing immunity. Peptides stimulate the proliferation of macrophages and lymphocytes through the NF-κB signaling pathway, thereby contributing to reducing inflammation and improving immunity. It is important to note that the mechanism by which peptides promote cell proliferation is not limited to signaling pathways. Next, we will explore the energy supply and proliferation cycle of cell proliferation, as well as the role of growth factors in promoting cell proliferation.

### 3.2. Regulation of Energy Metabolism

Cellular metabolism is responsible for providing energy for various life processes, including cell proliferation and movement during wound healing. It is crucial to maintain an adequate supply of adenosine triphosphate (ATP) for cellular restructuring, which relies on fluctuations in cellular energy demands [12]. Previous studies have demonstrated that the ATP/adenosine diphosphate (ADP) ratio serves as a reliable measure of intracellular energy consumption [78].

The oxygen consumption rate (OCR) was utilized to assess the impact of components on the mitochondrial respiratory chain through measures like ATP production, proton leak, maximal respiration, spare respiration, and basal respiration [12]. Cell proliferation-promoting peptide treatments may increase the mRNA expression levels of cyclooxygenase-2 (COX2), nuclear respiratory factor 1 (NRF-1), and mitochondrial transcription factor A (TFAM) to promote mitochondrial biogenesis [79].

### 3.3. Cell Cycle Regulation

Cell cycle regulation involves three dynamic biochemical processes known as G0/G1, S, and G2/M phases. The G0/G1 phases represent quiescent and early DNA synthesis stages, while the G2/M phases signify late DNA synthesis and mitosis stages. The DNA synthesis phase, S phase, represents active cell proliferation [80]. Studies have indicated that various signaling pathways and molecules are involved in regulating cell proliferation through mechanisms related to the cell cycle [81]. Sea cucumber-derived tetrapeptides VTPY and VLLY significantly increased the percentage of the S phase in the cell cycle of HUVEC via the upregulation of ERK and AKT signaling pathways. The VTPY and VLLY treatments effectively increased Cyclin D1 protein levels [12]. VGRTNSH derived from *Crassostrea hongkongensis* elevated the gene expression levels of the cell cycle protein CCND1. Treatment with peptide VGRTNSH induced significant progression from the G1 phase to the S phase in MCF-10A cells [38].

### 3.4. Regulation of Cytokines and Growth Factors

The principal cytokines and growth factors, such as insulin-like growth factor-1 (IGF-1); transforming growth factor-α (TGF-α); and TGF-β, EGF, and fibroblast growth factor (FGF), play critical roles in skeletal development [9] and wound healing [82]. EGF has been shown to significantly enhance the migration and proliferation of both fibroblasts [83] and osteoblasts [7]. FGF promotes angiogenesis, as well as cell migration and proliferation, thereby facilitating wound healing [84]. TGF-β can induce the secretion of extracellular matrix proteins and promote proliferation, migration, and angiogenesis [85]. Pilose antler peptides promote osteoblast proliferation and differentiation, accelerating bone formation. In MC3T3-E1 cells, EGF, heme oxygenase-1 (HO-1), epidermal growth factor receptor (EGFR), and nuclear factor erythroid 2-related factor 2 (Nrf-2) expression were obviously increased after the pilose antler peptide treatment [86]. Marine collagen peptides (MCPs) from the skin of *Nibea japonica* have also been found to promote the migration and proliferation of NIH-3T3 cells through the NF-κB signaling pathway. MCPs further increased the protein levels of specific growth factors, including FGF, TGF-β, VEGF, and EGF in NIH-3T3 cells [14].

Currently, peptides have been found to promote cell proliferation through the above five signaling pathways, which are now recognized as clear pathways that promote cell proliferation. Mitochondrial respiration provides enough energy for cell proliferation and differentiation [12]. Alteration of cell-cycle progression and increased expression of cyclins offer favorable conditions for cell proliferation [51]. Peptides involved in wound healing possess anti-inflammatory properties and scavenge free radicals and oxidative enzymes. Furthermore, peptides stimulate the secretion of various cytokines and growth factors, indirectly promoting cell proliferation [84]. However, the mechanism remains unclear. Whether other potential pathways affect cell proliferation remains to be further studied.

## 4. Safety and Regulatory Framework for Promoting Cell Proliferation Peptides

Despite growing health awareness and a booming nutraceutical market, the peptide industry still faces major challenges. For peptides that promote cell proliferation, animal studies are needed to assess safety before clinical trials. These studies help decide whether human testing is appropriate and guide clinical trial design [87]. However, it is crucial to emphasize that the primary evidence of safety must ultimately come from human clinical trials [88]. This is due to the limitations of animal models in accurately representing factors such as drug pharmacokinetics and pharmacology. When planning animal studies, factors like administration route, dose, duration, and species must be carefully chosen [89]. In addition, the effects of long-term peptide intake on different body systems require more research. It is also important to study how processing and storage affect peptide activity and availability during product development [90]. Due to the complex enzymes of the human digestive system, these peptides are likely to be degraded in the digestive tract [91]. Therefore, further clinical research is imperative for gaining a deeper understanding of their safety concerns, interactions with food matrices, bioavailability, and gastrointestinal stability before contemplating their utilization as functional foods or potential pharmaceuticals for prevention and treatment.

Regulatory concerns: the utilization of peptides that stimulate cell growth in food or other applications may require regulatory approval, which can be a time-consuming and expensive procedure [92]. With the growing popularity of health foods, misleading or false claims have become increasingly prevalent in the media. Therefore, establishing a clear regulatory framework for foods with bioactive peptides is essential to protect consumers from potential risks [93]. Different countries have varying regulatory systems. In China, all health food products must be registered and pre-approved by the CFDA. CFDA-authorized labs conduct tests on safety, function, stability, efficacy, and hygiene. The Health Food Expert Committee grants approval based on scientific data [88]. Approved products can be sold with the Blue Hat logo [94].

It is a long process, from discovering peptides that promote cell proliferation to their application in humans. Currently, peptides that promote cell proliferation have only been tested in vitro; further evaluations using animal models and clinical trials are necessary. The impact of food peptides on drug levels, allergenicity, processing, storage, and digestion requires further investigation [90]. Due to the limitations of animal models, clinical trials in humans are the only way to obtain reliable evidence. If food peptides are transformed into nutraceuticals or clinical medicinal products, their commercialization must adhere to market regulations and relevant laws. Registration and pre-approval by the CFDA are necessary before marketing and selling these products [88].

## 5. Discussion

Most peptides that promote cell proliferation from food sources are short or medium in size. Short peptides often show higher biological activity. They have various effects, such as relieving osteoporosis, promoting wound healing, and providing anti-inflammatory and antioxidant benefits. Cell proliferation is the foundation of these effects. Most peptides are extracted from aquatic animals, and many of them exhibit anti-osteoporotic activity. This is because osteoblast proliferation and differentiation help improve bone health and relieve osteoporosis. Notably, most peptides are extracted from aquatic animals. The peptides extracted from aquatic animals exhibit anti-osteoporotic effects. The proliferation and differentiation of osteoblasts play an important role in alleviating osteoporosis and promoting bone growth.

It should be noted that the unavailability of specific sequence information for certain source peptides, due to their exact sequences, has not been thoroughly characterized in the existing studies. While this limitation prevents a detailed structure-activity analysis, the observed results can still be interpreted as a composite effect of multiple peptide factors. Future studies should prioritize the sequencing and precise characterization of such peptides to enable more mechanistic insights. Until then, the conclusions drawn in their reports should be contextualized within these acknowledged constraints.

The functional activity of cell proliferation-promoting peptides depends not only on their sequence but also on factors such as digestion, processing, storage, and interactions with other components in the food matrix. Processing and storage methods contribute to product variability, which may alter the bioactivity of peptides. Moreover, other food substances may influence peptide function, a question that requires further investigation [90]. Once ingested, peptides undergo enzymatic digestion in the gastrointestinal tract, breaking down into smaller fragments. These smaller peptides may exhibit enhanced bioavailability, but their biological functions could also be weakened or lost [2]. Therefore, it is necessary to study how digestion affects peptide sequences and to identify bioactive peptides in diverse food sources [93]. Despite these challenges, peptides that promote cell proliferation show promising potential. Many peptide fragments exhibit unique functions not found in native proteins and can supplement essential amino acids while supporting tissue growth, repair, and immune regulation. Thus, continued efforts in functional food research and product development are important for transforming these peptides into beneficial health products [95].

Given the side effects of synthetic drugs, research is shifting toward natural sources. Insects are emerging as a promising protein source, and peptides derived from insect proteins show anti-osteoporotic, anti-inflammatory, and antihypertensive activities. It is therefore expected that insect-derived peptides may also promote cell proliferation. However, this field is still relatively new and underexplored [96]. With the expansion of the insect processing industry, large quantities of by-products (e.g., heads, skins, guts, and bones) are generated. Though often considered inedible, these residues contain up to 60% protein by dry weight and represent a valuable source of bioactive peptides [97]. Thus, exploring peptides from food processing by-products—especially those with cell proliferation-promoting potential—is recommended [96]. Although biosynthesis is less applied compared to natural extraction, with the identification of extracted mixed polypeptide components and the understanding of the functional mechanisms of single-structured polypeptides, the biosynthesis of polypeptides is also a promising method.

Despite challenges in developing functional foods, advances in technology and scientific research are crucial to unlocking this potential. Peptide synthesis methods and dosage may also influence biological activity and should be carefully evaluated during product development. At present, in the studies of food-derived peptides, the focus is mainly on obtaining high-concentration peptides after processing and extraction, while few studies have considered the changes in natural peptide components contained in plant and animal tissues during the extraction process, as well as whether natural peptides still play a role in biological processes such as cell proliferation, antioxidation, and immune response after processing has not received widespread attention. Meanwhile, whether the functions of these natural peptides are directly related to the different functions of peptides from various plant and animal sources requires further in-depth exploration.

At the same time, it is worth noting that not all natural products or substances from food sources are completely harmless to health. Pharmacokinetics would significantly affect the activity and action of peptides. In subsequent research, we also need to conduct comprehensive considerations, including pharmacokinetics and the potential side effects of high-concentration peptides. Additionally, regarding the functions and mechanisms of food-derived peptides and polypeptides, recent research primarily focuses on cells and model animals, while clinical trials on primates and humans are relatively few. Therefore, the key aspects in future studies are that the subsequent safety assessment should focus on the food-derived polypeptides in both live animal potential side effects and human safety assessment clinical research to support practical applications.

Over the past few decades, an increasing effort has been made to identify biopeptides that can promote cell proliferation. However, not all biological peptides can have a promoting effect. Some polypeptides have not shown significant effects in the research subjects, and these studies may not have been widely reported. In addition, the potential pro-tumorigenic risks of promoting cell proliferation should also be considered, which is critical for researchers, producers, and consumers in the field. This work summarizes the cell proliferation-promoting peptides obtained from aquatic animals, livestock products, and plants. It reviews several signaling pathways and the biological processes that peptides may affect in promoting cell proliferation, providing a reference for subsequent research on peptides that promote cell proliferation (Figure 1). Meanwhile, the future development direction of bioactive peptide resarch is speculated. Studies on the amino acid composition and structure of cell proliferation-promoting peptides, along with their function, are still in their early stages. Furthermore, the production of peptides that promote cell proliferation is a significant concern. Currently, the production of these peptides is still at the laboratory scale. The commercialization of food peptides necessitates strict compliance with market regulations and laws. Further advancements are necessary to scale up production to an industrial level.

## Figures and Tables

**Table 3:** Cell proliferation-promoting peptides derived from livestock products.

Sequence	Source	Cell/Animal Model	Amount Added	Action Pathway	Cell Performance	Animal Performance	References
FKSETKNLL	Bovine lactoferrin	MC3T3-E1 cell	200 μg/mL	MAPK signaling pathway	Cell proliferation and differentiation ↑	Osteoporosis ↓	[51]
VSEE	Duck Egg White	MC3T3-E1 cell	1 mM	Wnt/β-catenin signaling pathway	Cell proliferation, differentiation, and mineralization ↑	Osteoporosis ↓, dyslipidemia ↓	[6]
—	Porcine bone	MC3T3-E1 cell	0.5 mg/mL	PI3K/AKT signaling pathway	Cell proliferation and differentiation ↑, Cell cycle progression ↑	Osteoporosis ↓	[55]
GPAGPPGPIGNV	Yak bones	MC3T3-E1 cell	60.6 mg/mL	Wnt/β-catenin signaling pathway	Cell proliferation and differentiation ↑	Osteoporosis ↓	[56]
GPAGPSGPAGK, GPPGSPGPR	Bovine Gelatin	MC3T3-E1 cell	3 mg/mL	MAPK/ERK1/2 signaling pathway	Cell proliferation, differentiation, and mineralization ↑	Osteoporosis ↓ Osteoarthritis ↓	[30]
—	Whey protein	MC3T3-E1 cell	500 μg/mL	—	Cell proliferation, differentiation, and mineralization ↑	Osteoporosis ↓protects bones	[57]
ARHPHPHLSF, AAGGPGAPADPGRPTGY, NIPPLTQTPVVVPPFLQPE	Fermented milk	MC3T3-E1 cell	2 μM	MAPK signaling pathway	Cell proliferation, differentiation, and mineralization ↑	Osteoporosis ↓protects bones	[5]
HHGDQGAPGAVGPAGPRGPAGPSGPAGKDGR, GPAGANDRGEAGPAGPAGPR	Bovine Bone	MC3T3-E1 cell	48.0 mg/mL	—	Cell proliferation and differentiation ↑	Osteoporosis ↓	[54]
PASTGAAK, PGPPGTPF	black-boned silky fowl	MC3T3-E1 cell	400 μg/mL	BMP-2/Smad signaling pathway	Cell proliferation and differentiation ↑	Osteoporosis ↓	[58]
VLVLDTDYKK, VGINYWLAHK	Whey protein	RAW 264.7 cell	1.25 mg/mL	—	Cell proliferation ↑	Free radicals ↓, anti-inflammatory	[53]
COLPROPURD	Porcine fresh bones	Monocytic, lymphocyte, and Caco-2	0.15 mg/mL, 1.4 mg/mL,137.5 μg/mL	—	Cell proliferation ↑ and cytokine ↑	Anti-intestinal inflammation, immunomodulation ↑	[18]
EF, AGGF, EHPT	Black-bone silky fowl	Mice spleen	1 mM	—	Lymphocyte proliferation ↑	Immunomodulation ↑	[52]

↑: increase; ↓: decrease; —: reported but not fully confirmed.

## Data Availability

No new data were created or analyzed in this study.

## Abbreviations

The following abbreviations are used in this manuscript:

ECM	Extracellular matrix
HUVEC	Human umbilical vein endothelial cells
TOF	Time-of-flight
HPLC-MS/MS	High-performance liquid chromatography-tandem mass spectrometry
MSCs	Mesenchymal stem cells
BMSCs	Bone marrow-derived mesenchymal stem cells
OCR	Oxygen consumption rate
COX2	Cyclooxygenase-2
NRF-1	Nuclear respiratory factor 1
TFAM	Mitochondrial transcription factor A
AKT	Protein kinases (ERK) and protein kinase B
TGF-α	Transforming growth factor-α
MAPK	Mitogen-activated protein kinase
NF-κB	Nuclear factor κ-B
IKKα	IκB kinase α
FGF	Fibroblast growth factor
EGF	Epidermal growth factor
VEGF	Vascular endothelial growth factor A
MCPs	Marine collagen peptides
CFDA	China Food and Drug Administration
IGF-1R	Insulin-like growth factor 1 receptor
ERK1/2	Extracellular regulated protein kinases 1/2
SMAD	Mothers against decapentaplegic
PI3K	Phosphoinositide 3-kinase
LRP1	LDL receptor-related protein 1
PGE2	Prostaglandin E2
IL-6	Interleukin-6
BMP-2	Bone morphogenetic protein type 2
S6KB1	Ribosomal protein S6 kinase B1
mTOR	Mammalian target of rapamycin
TLR	Toll-like receptors
RAW264.7	Mouse mononuclear macrophage cells
MC3T3-E1	Mouse embryo osteoblast precursor cells
mTORC1/mTORC2	Mechanistic target of rapamycin complex 1/2
HaCaT	Human immortalized epidermal cells
JNK	c-Jun N-terminal kinase
ERK	Extracellular-regulated protein kinases
AP-1	Activator protein 1
MyoD	Myogenic differentiation antigen
MyoG	Myogenin
MyHC	Myosin heavy chain
GSK3β	Glycogen synthase kinase 3 beta
LRP5/6	Low-density lipoprotein receptor-related proteins 5 and 6
UPLC	Ultra-Performance Liquid Chromatography
TCF/LEF	T cell factor/lymphoid enhancer factor family
LRP3	Low-density lipoprotein receptor-related protein 3
IκB	Inhibitor of NF-κB
ROS	Reactive oxygen species
MEK	Mitogen-activated extracellular signal-regulated kinase
PIP3	Phosphatidylinositol-3,4,5-trisphosphate
PTEN	Phosphatase and tensin homolog
GAB1	GRB2-associated binder 1
PDK1	Pyruvate dehydrogenase kinase 1
GSK-3	Glycogen synthase kinase-3
BMP	Bone morphogenetic protein
ATP	Adenosine triphosphate
MCF-10A	Human normal breast cells
HO-1	Heme oxygenase-1
EGFR	Epidermal growth factor receptor
Nrf-2	Nuclear factor erythroid 2-related factor 2
NIH-3T3	Mouse embryonic fibroblast cells
TNF-α	Tumor necrosis factor alpha
TGF-β	Transforming growth factor-β
MG-63	Human osteosarcoma cell
HSF	human skin fibroblast
BMMS	Bone marrow-derived macrophage cell
hDPC	Human dermal papilla cells
IEC-6	Intestine crypt epithelial cell
CEF	Chicken embryonic fibroblast cell
GSK-3β	Glycogen synthase kinase 3 beta
IKKβ	IκB kinase β
PIP2	Phosphatidylinositol 4,5-bisphosphate
BMP-2/4	Bone morphogenetic proteins-2/4
SMAD1/5	Mothers against decapentaplegic homolog 1/5
BMP-2/4	Bone morphogenetic protein 2/4
ADP	Adenosine diphosphate
IGF-1	Insulin-like growth factor-1
